# The Complete Genome Sequence of *Pseudomonas putida* NBRC 14164^T^ Confirms High Intraspecies Variation

**DOI:** 10.1128/genomeA.00029-14

**Published:** 2014-02-13

**Authors:** Shoko Ohji, Atsushi Yamazoe, Akira Hosoyama, Keiko Tsuchikane, Takayuki Ezaki, Nobuyuki Fujita

**Affiliations:** a Biological Resource Center, National Institute of Technology and Evaluation, Shibuya-ku, Tokyo, Japan; b Department of Microbiology, Gifu University Graduate School of Medicine, Gifu, Japan

## Abstract

*Pseudomonas putida* has attracted much interest for its environmental, industrial, biotechnological, and clinical importance. Here, we report the complete genome sequence of the type strain *P. putida* NBRC 14164. This genome sequence will assist to further elucidate the molecular mechanisms of the characteristic traits among strains belonging to the species *P. putida*.

## GENOME ANNOUNCEMENT

*Pseudomonas putida* is isolated from a wide range of ecological niches, which reflects its ability to adapt to different environmental conditions, and it shows its versatile metabolic capacity (1, 2). It has been also found to be an opportunistic human pathogen (3). Despite the increasing number of projects that are sequencing this species, the genome of the type strain has not been sequenced (http://www.genomesonline.org/).

The genome of *P. putida* type strain NBRC 14164 was sequenced by using a combined strategy of shotgun sequencing with 454 GS FLX Titanium (Roche) (106.9-Mb sequences, 17.4-fold coverage) and paired-end sequencing with HiSeq 1000 (Illumina) (572.6-Mb sequences, 93.0-fold coverage). The 454 Titanium and Illumina paired-end data were assembled with Newbler version 2.3. Genome closure was accomplished by additional Sanger sequencing of targeted PCR products and fosmid clones. The complete sequence of the chromosome was analyzed using the Microbial Genome Annotation Pipeline (MiGAP) (http://www.migap.org/) for predicting protein-coding, tRNA, and rRNA genes. The functional annotations of the predicted protein-coding genes were assigned based on UniProt, InterPro, HAMAP, and an in-house database composed of manually curated microbial genome sequences, as reported previously (4).

The genome of strain NBRC 14164 consists of a 6,156,701-bp circular chromosome with a 62.3% G+C content and contains 5,449 coding sequences (CDSs), 7 copies of rRNA operons, and 74 tRNA genes. A pairwise bidirectional BLASTp analysis of the CDSs was performed between NBRC 14164 and each of 9 other genome-sequenced *P. putida* strains (KT2440, GB-1, W619, BIRD-1, S16, F1, PC9, UW4, and ND6). It showed that the 10 strains share 3,031 CDSs and NBRC 14164 contains 469 unique CDSs (no BLAST hit to any CDS in the other 9 strains, with a threshold e-value of 10^-5^). The closest relative to NBRC 14164 is *P. putida* strain GB-1, sharing 1,485 additional genes.

A calculation of the average nucleotide identity (ANI) was also performed between the genome sequences of the 10 strains using the JSpecies program with default settings for ANI based on BLAST (ANIb) (http://www.imedea.uib.es/jspecies). The ANI values of type strain NBRC 14164 against the other 9 strains of *P. putida* range from 77.26% (with strain UW4) to 90.36% (with strain GB-1), showing lower values than the 95 to 96% threshold used to distinguish bacterial species (5, 6). This result suggests that the 9 sequenced strains might be taxonomically different from *P. putida*.

The genome of strain NBRC 14164 contains 111 out of 453 known virulence factor genes in the virulence factors database for *Pseudomonas* (http://www.mgc.ac.cn/VFs/), including genes responsible for the synthesis of flagella, type IV pili, alginate, and pyoverdine. However, the genome is missing complete gene sets for a type III secretion system and associated effector proteins.

This genome sequence of NBRC 14164 will serve as a valuable reference for understanding the genetic characteristics, exploring industrial potentials, and addressing the clinical challenges of *P. putida* and related species.

### Nucleotide sequence accession number.

The nucleotide sequence of the *P. putida* NBRC 14164 chromosome was deposited in the DDBJ/EMBL/GenBank databases under the accession no. AP013070.

## References

[1] Wu X Monchy S Taghavi S Zhu W Ramos J van der Lelie D 2011 Comparative genomics and functional analysis of niche-specific adaptation in *Pseudomonas putida*. FEMS Microbiol. Rev. 35:299–323. 10.1111/j.1574-6976.2010.00249.x 20796030 PMC3056050

[2] Poblete-Castro I Becker J Dohnt K dos Santos VM Wittmann C 2012 Industrial biotechnology of *Pseudomonas putida* and related species. Appl. Microbiol. Biotechnol. 93:2279–2290. 10.1007/s00253-012-3928-0 22350258

[3] Yoshino Y Kitazawa T Kamimura M Tatsuno K Ota Y Yotsuyanagi H 2011 *Pseudomonas putida* bacteremia in adult patients: five case reports and a review of the literature. J. Infect. Chemother. 17:278–282. 10.1007/s10156-010-0114-0 20809240

[4] Shintani M Hosoyama A Ohji S Tsuchikane K Takarada H Yamazoe A Fujita N Nojiri H 2013 Complete genome sequence of the carbazole degrader *Pseudomonas resinovorans* strain CA10 (NBRC 106553). Genome Announc. 1(4):e00488-13. 10.1128/genomeA.00488-13 23887915 PMC3735057

[5] Goris J Konstantinidis KT Klappenbach JA Coenye T Vandamme P Tiedje JM 2007 DNA-DNA hybridization values and their relationship to whole-genome sequence similarities. Int. J. Syst. Evol. Microbiol. 57:81–91. 10.1099/ijs.0.64483-0 17220447

[6] Richter M Rosselló-Móra R 2009 Shifting the genomic gold standard for the prokaryotic species definition. Proc. Natl. Acad. Sci. U. S. A. 106:19126–19131. 10.1073/pnas.0906412106 19855009 PMC2776425

